# Single-cell mass cytometry of microglia in major depressive disorder reveals a non-inflammatory phenotype with increased homeostatic marker expression

**DOI:** 10.1038/s41398-020-00992-2

**Published:** 2020-09-11

**Authors:** Chotima Böttcher, Camila Fernández-Zapata, Gijsje J. L. Snijders, Stephan Schlickeiser, Marjolein A. M. Sneeboer, Desiree Kunkel, Lot D. De Witte, Josef Priller

**Affiliations:** 1 Department of Neuropsychiatry and Laboratory of Molecular Psychiatry, Charité – Universitätsmedizin Berlin, Berlin, Germany; 2 Department of Psychiatry, Brain Center Rudolf Magnus, University Medical Center Utrecht, Utrecht, The Netherlands; 3 BIH Center for Regenerative Therapies (BCRT), Charité – Universitätsmedizin Berlin, Berlin, Germany; 4 Department of Psychiatry, Icahn School of Medicine at Mount Sinai, New York, NY USA; 5 Flow & Mass Cytometry Core Facility, Charité – Universitätsmedizin Berlin and Berlin Institute of Health (BIH), 10178 Berlin, Germany; 6 Mental Illness Research, Education and Clinical Center (MIRECC), James J Peters VA Medical Center, Bronx, NY USA; 7 DZNE and BIH, Berlin, Germany; 8 University of Edinburgh and UK Dementia Research Institute (DRI), Edinburgh, UK

**Keywords:** Molecular neuroscience, Depression

## Abstract

Stress-induced disturbances of brain homeostasis and neuroinflammation have been implicated in the pathophysiology of mood disorders. In major depressive disorder (MDD), elevated levels of proinflammatory cytokines and chemokines can be found in peripheral blood, but very little is known about the changes that occur directly in the brain. Microglia are the primary immune effector cells of the central nervous system and exquisitely sensitive to changes in the brain microenvironment. Here, we performed the first single-cell analysis of microglia from four different post-mortem brain regions (frontal lobe, temporal lobe, thalamus, and subventricular zone) of medicated individuals with MDD compared to controls. We found no evidence for the induction of inflammation-associated molecules, such as CD11b, CD45, CCL2, IL-1β, IL-6, TNF, MIP-1β (CCL4), IL-10, and even decreased expression of HLA-DR and CD68 in microglia from MDD cases. In contrast, we detected increased levels of the homeostatic proteins P2Y_12_ receptor, TMEM119 and CCR5 (CD195) in microglia from all brain regions of individuals with MDD. We also identified enrichment of non-inflammatory CD206^hi^ macrophages in the brains of MDD cases. In sum, our results suggest enhanced homeostatic functions of microglia in MDD.

## Introduction

Major depressive disorder (MDD) is one of the most common mental disorders across the lifespan and represents a leading cause of disability worldwide^1^. MDD is more prevalent in women than men and it increases the risk of suicide, obesity, and coronary heart disease^2^. Recent genome-wide association studies have associated MDD with variants in genes involved in hypothalamic-pituitary-adrenal (HPA) axis function, neuronal differentiation, synaptic transmission, cytokine production, and immune response^3^. Cell type-specific methylome studies have confirmed the involvement of the innate immune system in MDD^4^. The polygenic risk for MDD is moderated by environmental factors, such as childhood trauma^5^. Strong gene-environment interactions exist between HPA axis genes (*Corticotrophin releasing hormone receptor 1*, *FK506 binding protein 5*) and MDD^6^. Notably, higher *FKBP5* expression promotes nuclear factor (NF)-κB-mediated peripheral inflammation and chemotaxis^7^. Along these lines, chronic stress and low-grade inflammation are believed to contribute to the pathophysiology of MDD^8–10^. In fact, patients with MDD express increased levels of proinflammatory cytokines like interleukin (IL)-1, IL-6 and tumor necrosis factor (TNF)-α in peripheral blood, which can access the central nervous system (CNS) and activate tissue-resident macrophages like microglia, impair HPA axis function, modulate monoaminergic neurotransmission, and reduce neural plasticity^11–13^. The tryptophan-kynurenine pathway links depression and inflammation, and under stressful and inflammatory conditions, microglial indoleamine 2,3-dioxygenase (IDO) activity reduces serotonin availability and results in the production of excitotoxic metabolites such as quinolinic acid^8,9^. In recent years, peripheral blood cytokines have been proposed as biomarkers of MDD^14^, and combinations of pro- and anti-inflammatory cytokines (e.g. IL-10) can help predict the response to antidepressant treatment^15–18^.

However, it remains an unresolved question of high therapeutic relevance whether the inflammation in MDD originates primarily in the periphery or in the CNS. Peripheral blood monocytes of MDD patients express higher levels of inflammatory/immune mediators like IL-1β and IL-6, as well as *TNF*, *TLR2*, *CEBPA,* and *CCL2* mRNAs^19–24^. In contrast, studies of CNS microglia have resulted in contradictory findings. Microglia are specialized tissue macrophages that originate from the yolk sac during early embryonic life, and play essential roles in brain development and maintenance of CNS homeostasis^25^. Neuropathological examination of post-mortem frontal lobe tissue from MDD cases revealed increased numbers of activated microglia expressing ionized calcium-binding adaptor molecule (Iba)1^26^ or quinolinic acid^27^. The number of primed Iba1-positive microglia and CD45-immunoreactive perivascular macrophages was increased in depressed suicides^28^. In contrast, many other studies did not detect significant changes in the density of major histocompatibility complex HLA-DR-immunoreactive microglia in frontal lobe, temporal lobe, thalamus or brain stem of MDD cases^29–32^. Gene expression profiling of post-mortem frontal lobe tissue from psychotropic drug-free persons with a history of MDD revealed increased expression of *IL1A*, *IL3*, *IL5*, *IL8*, *IL10*, but not *IL6* or *TNF*^33^. Gene expression of *TNF*, *IFNG,* and *CCL2* was even reduced in the prefrontal cortex of depressed suicides^28^. Strong support for the neuroinflammation hypothesis of MDD comes from positron emission tomography (PET) studies of translocator protein 18 kDa (TSPO) binding in depressed individuals with MDD. TSPO binding was found to be elevated in frontal lobe, temporal lobe and thalamus of depressed patients, and correlated with the severity of depression^34^, cognitive dysfunction^35^, and the duration of antidepressant treatment^36^. However, no correlation with peripheral inflammatory markers like IL-1β, IL-6, TNF, and C-reactive protein (CRP) was detected^34^. It is also still controversial whether TSPO binding is a good indicator of microglial activation in the human brain^37^.

Given that the role of microglia is not yet clear in major depression^38^, we decided to use single-cell high-dimensional mass cytometry (CyTOF) to examine microglia from different post-mortem brain regions of medicated individuals with MDD compared to controls. We have recently demonstrated that this technique allows us to detect subtle phenotypic differences of human microglia across different brain regions with good correlation between post-mortem tissue and fresh brain biopsies^39^. In this study, we determined 59 protein markers at the single-cell level that unequivocally distinguish microglia from other brain macrophages and peripherally derived immune cells. The results suggest a non-inflammatory phenotype of microglia with increased homeostatic marker expression in MDD.

## Methods

### Human post-mortem tissue

Human post-mortem brain tissue was obtained from the Psychiatric Donor Program of the Netherlands Brain Bank (NBB-Psy; www.brainbank.nl). The Netherlands Brain Bank received permission to perform autopsies and to use tissue and medical records from the Ethical Committee of the VU University Medical Center. Tissue was collected post-mortem from donors from whom full consent had been obtained during life to conduct brain autopsy and research. Mediacted MDD cases (*n* = 6) were defined as donors with a diagnosis of MDD according to the DSM-IV or III. Control donors (*n* = 5) were defined as donors without a history of depression, confirmed by retrospective medical chart review. Detailed donor information is provided in Supplementary Tables 1–3.

### Microglia isolation

Microglia were isolated from post-mortem brain tissue as described previously^39^. After autopsy, tissue was stored in Hibernate medium (Invitrogen, Carlsbad, CA, USA) at 4 °C until further processing. Microglia isolation started as soon as possible, at the latest after 24 h. A single-cell suspension was generated by mechanical and enzymatic digestion with collagenase (3700 units/mL; Worthington, USA) and DNase (200 µg/mL; Roche, Switzerland) for frontal lobe (GFM), temporal lobe (GTS) and thalamic (THA) tissues, or 0.2% trypsin and 30 mg DNase for subventricular zone (SVZ) tissue. A Percoll (Amersham, Merck, Germany) gradient was generated to separate viable cells from myelin, cellular debris, and erythrocytes. The middle layer was collected and washed twice, followed by positive selection of myeloid cells with CD11b-conjugated magnetic beads (Miltenyi Biotec, Germany) according to the manufacturer’s protocol. MACS-isolated CD11b^+^ cells were fixed with fixation/stabilization buffer (SmartTube) and frozen at −80 °C until analysis by mass cytometry^39^.

### Intracellular barcoding for mass cytometry

MACS-isolated CD11b^+^ cells were thawed and subsequently stained with premade combinations of six different palladium isotopes: ^102^Pd, ^104^Pd, ^105^Pd, ^106^Pd, ^108^Pd, and ^110^Pd (Cell-ID 20-plex Pd Barcoding Kit, Fluidigm). This multiplexing kit applies a 6-choose-3 barcoding scheme that results in 20 different combinations of three Pd isotopes. After 30 min staining at room temperature, individual samples were washed twice with cell staining buffer (0.5% bovine serum albumin in PBS containing 2 mM EDTA). All samples were pooled together, washed, and stained with antibodies.

### Antibodies

Anti-human antibodies (Supplementary Tables 4 and 5) were purchased either pre-conjugated to metal isotopes (Fluidigm), or from commercial suppliers in purified form and then conjugated by us using the MaxPar X8 kit (Fluidigm) according to the manufacturer’s protocol. Each antibody was titrated and validated using different cell types from different body compartments, as described previously^39^.

### Cell surface and intracellular staining

After cell barcoding, washing and pelleting, the combined samples were stained and processed as described previously^39^. Briefly, cells were resuspended in 100 µl of antibody cocktail directed against cell surface markers (Supplementary Tables 4 and 5) and incubated at 4 °C for 30 min. Then, the cells were washed twice with cell staining buffer (PBS containing 0.5% bovine serum albumin and 2mM EDTA). For intracellular staining, the stained (non-stimulated) cells were incubated in fixation/permeabilization buffer (Fix/Perm Buffer, eBioscience) at 4 °C for 60 min. After two washes with permeabilization buffer (eBioscience), the samples were stained with antibody cocktails directed against intracellular molecules (Supplementary Tables 4 and 5) in permeabilization buffer at 4 °C for 1 h. Cells were subsequently washed twice with permeabilization buffer and incubated overnight in 4% methanol-free formaldehyde solution. The fixed cells were washed and resuspended in 1 ml iridium intercalator solution (Fluidigm) at room temperature for 1 h, followed by two washes with cell staining buffer and two washes with ddH_2_O (Fluidigm). Finally, cells were pelleted and kept at 4 °C until CyTOF measurement.

### Bead staining

For the bead-based compensation of the signal spillover, AbC total antibody compensation beads (Thermo Fisher Scientific) were stained with each of the antibodies used in all three antibody panels according to the manufacturer’s instructions. Stained beads were then measured with CyTOF and the compensation matrix was generated as described previously^40^.

### CyTOF measurements

Cells were analyzed using a CyTOF2 upgraded to Helios specifications, with software version 6.7.1014^39^. The instrument was tuned according to the manufacturer’s instructions with tuning solution (Fluidigm), and measurement of EQ four element calibration beads (Fluidigm) containing ^140/142^Ce, ^151/153^Eu, ^165^Ho, and ^175/176^Lu served as a quality control for sensitivity and recovery. Immediately prior to analysis, cells were resuspended in ddH_2_O, filtered through a 20 µm cell strainer (Celltrix, Sysmex), counted and adjusted to 3–5 × 10^5^ cells/ml. EQ four element calibration beads were added at a final concentration of 1:10 v/v in order to normalize the data to compensate for signal drift and day-to-day changes in instrument sensitivity. Samples were acquired with a flow rate of 300–400 events/s. The lower convolution threshold was set to 400, with noise reduction mode turned on and cell definition parameters set at event duration of 10–150 pushes (push = 13 µs). The resulting flow cytometry standard (FCS) files were normalized and randomized using the CyTOF software’s internal FCS-Processing module on the non-randomized (“original”) data. The default settings in the software were used with time interval normalization (100 s/minimum of 50 beads) and passport version 2. Intervals with less than 50 beads per 100 s were excluded from the resulting FCS file.

### Mass cytometry data processing and analysis

Following the workflow from our previous study^40^, Cytobank (www.cytobank.org) was used for initial manual gating on live single cells and Boolean gating for de-barcoding. Nucleated single intact cells were manually gated according to the signals of DNA intercalators ^191^Ir/^193^Ir and event length. For de-barcoding, Boolean gating was used to deconvolute individual samples according to the barcode combination. Prior to data analysis, each FCS file was compensated for signal spillover using R package *CATALYST*^41^. For dimensionality reduction, visualization and further exploration, (2D)-tSNE maps were generated based on the expression levels of all markers in each panel. For embedding, we set hyperparameters to perplexity of 30, theta of 0.5, and iterations of 1000 per 100,000 analyzed cells. To visualize marker expression, arcsinh transformation was applied to the data. All FCS files were then loaded into R and further data analysis was performed with a custom written script based on the workflow proposed by Nowicka and colleages^42^. Briefly, for unsupervised cell population identification, we performed cell clustering with the *FlowSOM*^43^ and *ConsensusClusterPlus*^44^ packages using all markers in each panel. We then performed visual inspection of cluster-colored tSNE plots and phenotypic heat maps for a more detailed profile of each cluster, and we determined the number of meta-clusters with consistent phenotypes for statistical testing. Based on visual inspection of t-SNE plots and heat maps generated at the merging step, a final number of meta-clusters was chosen that merged clusters into populations with consistent phenotypes (with a minimal mean frequency of 0.1% of parent)^40^.

### Statistical analysis

No randomization and blinding strategies were applied in this study. However, data processing and analysis, as well as statistical testing were carried out in an unsupervised manner. Dichotomous variables of the sample cohort were analyzed with Fisher’s exact test (GraphPad Prism). Quantitative data are shown as independent data points with Box-Whisker. Exploratory analyses of statistical significance were performed using multiple *t*-test available through GraphPad Prism 8 with a false discovery rate (FDR) adjustment at 10% using the Benjamini–Krieger–Yekutieli procedure for multiple hypothesis testing, unless otherwise stated. A *P* value < 0.05 was considered statistically significant.

## Results

### Samples

MDD and controls did not differ in age, gender or post-mortem characteristics (Supplementary Table 1). The average post-mortem delay was 8 h (range 4½–12¾ h). Both groups were medicated, and donors differed with regard to medication used and psychiatric history (Supplementary Tables 2 and 3).

### Regional diversity of human microglia is preserved in MDD

**Fig. 1:**
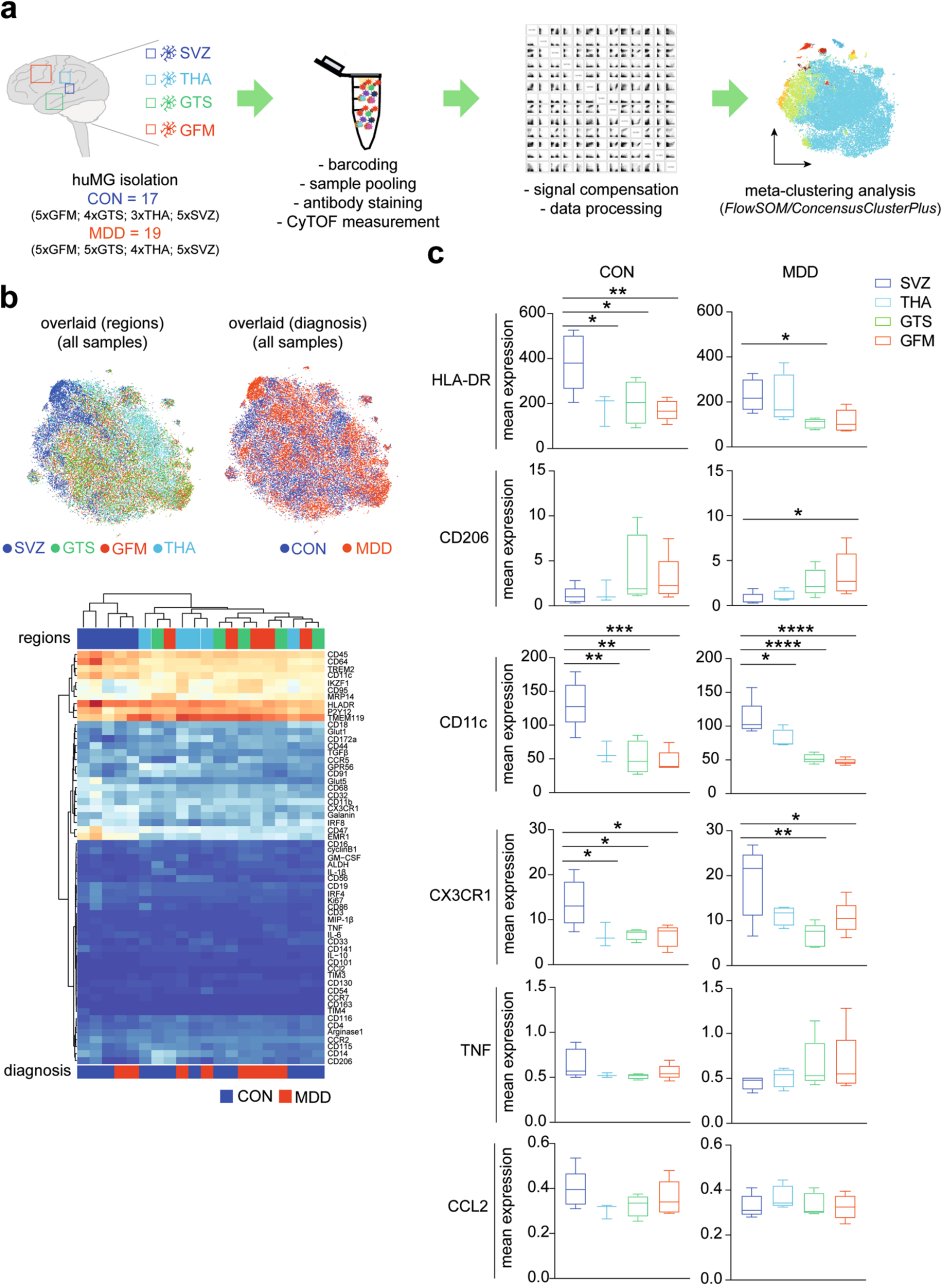
Regional heterogeneity of microglia. **a** Schematic representation of the experimental workflow for CyTOF. Human microglia (huMG) were isolated from the subventricular zone (SVZ, *n* = 10), thalamus (THA, *n* = 7), temporal lobe (GTS, *n* = 9), and frontal lobe (GFM, *n* = 10) of eleven independent donors (CON, *n* = 5; MDD, *n* = 6). HuMG samples were barcoded and pooled. Three different measurements of three different pooled samples were performed. Each pooled sample was divided in half and stained with two panels of metal-conjugated antibodies (*Panels A* and *B*, Supplementary Tables 4 and 5) and measured on the CyTOF instrument. Prior to algorithm-based data analysis, the data were demultiplexed and compensated. Clustering analysis was performed to discover small phenotypic differences between the studied groups using algorithm-based data analysis workflow, *FlowSOM/ConsensusClusterPlus*. **b** The overlaid t-SNE plot of 16 samples (SVZ = 4; THA = 4, GTS = 4; GFM = 4 from two biologically independent CON donors and two biologically independent MDD donors) is shown. The coloring denotes different regions (left image) and studied groups (right image). The 2D t-SNE maps were generated based on expression levels of all markers of *Panel A* (Supplementary Table 4). The heat map cluster (bottom) demonstrates the expression levels of 59 analyzed markers. Samples are indicated by dendrograms, regions and diagnosis are color-coded as above. Heat colors show overall expression levels (dark blue: no expression; red: high expression). **c**) Boxplots show mean expression levels of selected markers in different brain regions for huMG from CON and MDD cases. Boxes extend from the 25th to 75th percentiles. Whisker plots show the min (smallest) and max (largest) values. The line in the box denotes the median. *P < 0.05, **P < 0.01, ***P < 0.001, ****P < 0.0001, one-way ANOVA testing with Dunnett correction for multiple comparisons. All brain regions were compared with the SVZ.

Human microglia (huMG) were isolated from post-mortem brain tissue of subventricular zone (SVZ), thalamus (THA), temporal lobe (GTS) and frontal lobe (GFM) (Fig. 1a). In order to minimize the run-to-run variation and to facilitate the comparison of cellular profiles from different brain regions and individuals, we simultaneously profiled huMG samples from different brain regions of donors with MDD and controls in the same run. To do so, huMG were intracellularly barcoded using different combinations of palladium isotopes as described previously^39^. Up to twenty samples were pooled, split equally and stained with two different antibody panels (Supplementary Tables 4 and 5). The antibody *Panel A* (36 antibodies) was designed to distinguish the major circulating immune cell subsets, including T & B lymphocytes, monocytes, natural killer (NK) cells, from CNS immune cells, including microglia and border-associated macrophages. The panel also focused on cytokines, chemokines and other inflammatory mediators, and comprised antibodies against P2Y_12_ receptor, TREM2, CD45, CD3, CD14, CD16, CD11c, CD64, CD11b, CD56, EMR1, CD115, CD47, CD19, HLA-DR, CD56, CD68, CD33 (Siglec-3), CD192 (CCR2), CD195 (CCR5), CX3CR1, CD141, CD32, CD206, CD163, MIP-1β, IL-10, CCL2, IRF8, TGFβ, and TNF (Supplementary Table 4). The antibody *Panel B* was designed to investigate functional and activity changes in huMG subsets using 35 antibodies, including HLA-DR, IKZF1, ALDH, IL-1β, IL-6, MRP14 (S100A9), CD11b, CD116, CD44, Galanin, CD54 (ICAM1), CCR7, GPR56, CD141, CD86, CD91 (LRP1), CD95, CD172a, Glut1, Glut5, TIM3, TIM4, Arginase-1 (Supplementary Table 5). Multiplexed and stained samples were simultaneously acquired on a CyTOF instrument. To validate the robustness of the results, we performed three independent measurements with a total of 36 huMG samples (summarized in Supplementary Table 1).

We used the commercially available analysis platform Cytobank (www.cytobank.org) to capture and visualize all huMG cells in a single two-dimensional (2D) map using unsupervised high-dimensional data analysis, the t-distributed stochastic linear embedding (t-SNE) algorithm (Fig. 1b). In line with our previous evidence for regional heterogeneity of human microglia^39^, we observed a unique phenotype of SVZ microglia compared to microglia isolated from other brain regions (Fig. 1b, c). The huMG from control SVZ showed significantly higher expression of HLA-DR, CD11c, and CX3CR1 (Fig. 1c). Most importantly, we did not observe separation of huMG from donors with MDD and controls (Fig. 1b), which is in strong contrast to what we had previously observed for glioma-associated huMG^45^. No differences in the expression of HLA-DR, CD206, CD11c, CX3CR1, TNF, and CCL2 were observed for huMG from different brain regions between control and MDD cases (Fig. 1c). However, huMG from MDD cases expressed attenuated levels of HLA-DR in SVZ (Fig. 1c).

### Increased P2Y_12_ and TMEM119 expression in microglia from MDD brains

**Fig. 2:**
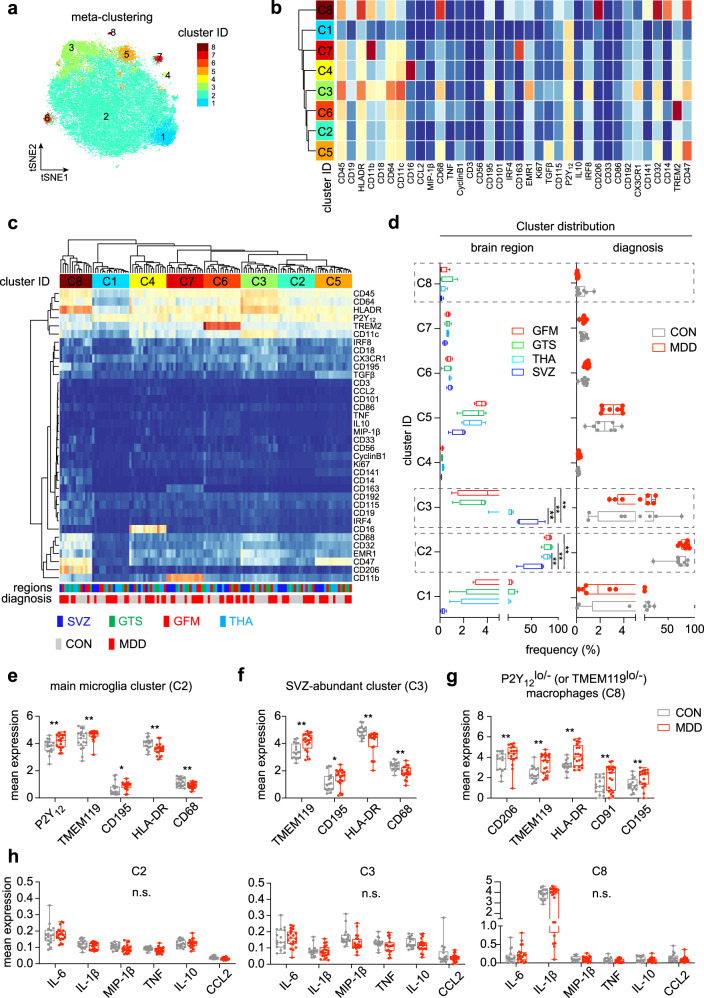
Phenotypic diversity of myeloid cells in the MDD brain. **a** The overlaid t-SNE plot of 16 huMG samples (SVZ = 4; THA = 4; GTS = 4; GFM = 4) isolated from four biologically independent donors (CON = 2; MDD = 2) is shown. The 2D t-SNE maps were generated based on expression levels of all markers of *Panel A* (Supplementary Table 4). The coloring indicates eight defined clusters representing diverse myeloid cell phenotypes. **b** Heat map and cluster analysis of all samples demonstrates the phenotypes of all eight defined clusters on the basis of the mean expression levels of 36 markers used for the cluster analysis. Identified clusters are indicated by dendrograms. Heat colors show overall marker expression levels (red: high expression; dark blue: no expression). **c** Heat map cluster demonstrates the expression levels of 36 analyzed markers for all eight clusters of each sample. Samples are indicated by dendrograms. Heat colors show overall expression levels (dark blue: no expression; red: high expression). **d** Boxplots on the left show the cluster distribution across four different brain regions (SVZ, blue; THA, light blue; GTS, green; GFM, red) and on the right the cluster distribution for the two groups (CON and MDD). Whisker plots show the min (smallest) and max (largest) values. A dot indicates the frequency (%) of an individual sample. The line in the box denotes the median. **P < 0.01, multiple t-test with FDR adjustment (at 10% using the Benjamini–Krieger–Yekutieli procedure). (**e**–**g**) Boxplots showing markers with differential expression (arcsinh) between CON and MDD for (**e**) the main microglia cluster C2, (**f**) the SVZ-enriched huMG cluster C3, and (**g**) the P2Y_12_^lo/−^ (or TMEM119^lo/−^) macrophage cluster C8. A dot indicates the mean expression of an individual sample from all three measurements. *P < 0.05, **P < 0.01, multiple t-test with FDR adjustment (at 10% using the Benjamini-Krieger-Yekutieli procedure). **h** Boxplots showing selected cytokines and chemokines (IL-6, IL-1β, MIP-1β, TNF, IL-10, CCL2) that are not differentially expressed (arcsinh) between CON and MDD for the clusters C2, C3, and C8. A dot indicates the mean expression of an individual sample from all three measurements. A *P* value < 0.05 was considered statistically significant (multiple t-test with FDR adjustment at 10% using the Benjamini–Krieger–Yekutieli procedure).

Next, to fully harness the high-dimensionality of the mass cytometry data and to study huMG in more detail, we performed a comprehensive analysis on R/Bioconductor using the over-clustering approach^40^. Of note, the number of defined clusters may not solely represent biological functional subsets of huMG, but should be considered as an exploratory tool for the discovery of differential abundance of small/rare huMG populations between the two analyzed groups. All markers of antibody *Panels A and B* were included in the meta-clustering analysis, revealing eight distinct phenotype clusters of huMG (Fig. 2a–d). The defined clusters displayed a differential distribution across the different brain regions (Fig. 2c, d). The main cluster 2 (C2) was significantly less abundant in SVZ, whereas cluster 3 (C3) was more abundant in SVZ (Fig. 2d). No significant differences in cluster distribution were observed between huMG from MDD and control cases (Fig. 2d).

Comparison of microglia phenotypes within defined clusters revealed significant differences between MDD and control cases (Fig. 2e–g). Notably, the main cluster C2 contained huMG with significantly higher expression of the homeostatic markers P2Y_12_ and transmembrane protein (TMEM)119^46^ in MDD compared to controls (Fig. 2e). At the same time, huMG from cluster C2 expressed lower levels of the activation markers HLA-DR and CD68^47^ in MDD (Fig. 2e). HuMG from the SVZ-enriched cluster C3 also expressed more TMEM119 and less HLA-DR and CD68 in MDD cases (Fig. 2f). In contrast, CD206^hi^ (P2Y_12_^lo^TMEM119^lo^) macrophages from cluster C8 expressed more HLA-DR, TMEM119 and CD91 (LRP1) in MDD than control cases (Fig. 2g). Importantly, huMG/macrophages from clusters C2, C3, and C8 expressed higher levels of the chemokine CD195 (CLL5), but comparable levels of IL-1β, IL-6, TNF, MIP-1β (CCL4), IL-10, and MCP-1 (CCL2) between MDD and control cases (Fig. 2e–g). None of the other examined markers from *Panels A and B*, including among others CX3CR1, IRF8, IRF7, CD11b, CD11c, CD86, CD45, TREM2, APOE, AXL, CD33 (Siglec-3), CD54 (ICAM1), CD56 (NCAM), GPR56, CD74, TGFβ, IFN-α, Galanin, CD95 (Fas), PD-L1, and Glut5, were differentially expressed between huMG/macrophages from MDD and control cases (data not shown).

## Discussion

This is the first study to examine human microglia in MDD at the single-cell level. We found a non-inflammatory phenotype of microglia with enhanced homeostatic functions in medicated MDD cases.

Our results are in line with recent microarray-based transcriptomic profiling of cerebral cortex in 87 cases of MDD, revealing no significant changes (FDR-corrected *P* value > 0.05) in the gene expression of *HLA-DR*, *TNF*, *IL6*, *IL1B*, *IL1A*, *IL3*, *IL5*, *IL8*, *IL10*, *ITGAM* (*CD11b), FKBP5, TLR2*, *CCL2,* and *CD14* compared with matched controls^48^. Notably, the study detected increased transcript levels of *IL17* and *CCL5*, the latter binding to the chemokine receptor CCR5, which we found increased by CyTOF in human microglia from MDD brains. Interestingly, systemic inflammation can trigger CCR5-dependent migration of microglia to the cerebral vasculature^49^. Gene expression profiling of post-mortem frontal lobe tissue from 14 MDD cases showed increased expression of *IL1A*, *IL3*, *IL5*, *IL8*, *IL10*, but not *IL6* or *TNF*^33^. Gene expression of *TNF*, *IL1B* and *IL10* was also unchanged in the prefrontal cortex of depressed suicides^28^. We have performed PCR analysis of myeloid cells from post-mortem brain tissue of 20 MDD cases compared with 27 controls and found no differential expression of *IL6*, *IL1B*, and *TNF* mRNAs, even after in vitro challenge with lipopolysaccharide and dexamethasone (Snijders et al., submitted for publication). However, we detected increased expression of *CX3CR1* and *TMEM119* mRNAs, and decreased expression of *CD163* and CD14 protein (Snijders et al., submitted for publication), underscoring the results of this single-cell CyTOF analysis of human microglia in the MDD brain.

The power of high-dimensional single-cell analysis lies in the precise identification of cell populations and the detection of rare disease-associated microglia states that would go undetected with bulk analysis and the use of single markers^45,50–52^. Even though the sample size is very small and medication effects cannot be excluded, our study suggests that homeostatic functions may be enhanced in MDD microglia. It is tempting to speculate that the increased expression of TMEM119 and P2Y_12_ in microglia clusters from MDD cases may reflect enhanced neuron-microglia communication via transforming growth factor (TGF)β1, which shows significant gene-environment interactions predicting adult depression in the context of early life trauma^53^, as well as purines, in an attempt to protect neuronal function^54,55^. Along these lines, monocyte-derived microglia-like cells from individuals with schizophrenia exhibit increased synapse engulfment^56^. Expression of P2Y_12_ in microglia is important for synaptic plasticity^57^ and for adult hippocampal neurogenesis^58^, which has been associated with the response to antidepressant treatment^59^. Neuronal activity regulates the surveilling function of microglia processes in the cortex via the monoamine neurotransmitter norepinephrine^60,61^, which is reduced in the brains of patients with depression^62,63^. A reduction in norepinephrine tone results in microglial extension and territory surveillance^60^. Interestingly, microglia were found to be hyper-ramified in a chronic despair mouse model of depression, and this morphological change was mediated by neuron-microglia signaling via CX3CR1, and restored by antidepressant treatment^64^.

Neuroinflammation is associated with the marked downregulation of homeostatic markers like P2Y_12_ and TMEM119 in microglia^52,65,66^. In contrast, we found that both markers were increased in microglia from MDD brains and none of the proinflammatory mediators were altered, calling into question the presence of an active inflammatory process. In fact, we even detected a downregulation of the immune molecules, HLA-DR and CD68, in the main cluster of microglia from MDD brains compared with controls. HLA-DR is a major histocompatibility complex (MHC) class II molecule involved in antigen presentation, which is constitutively expressed by human microglia with higher expression levels in white than gray matter^45,67^. Expression of HLA-DR is strongly induced in activated microglia across a variety of neuroinflammatory and neurodegenerative diseases^52,68^, and axonal damage in multiple sclerosis has been associated with HLA-DR^+^ microglia^69^. CD68 is a glycoprotein that primarily localizes to the endosomal/lysosomal compartment, but also acts as a class D scavenger receptor on the plasma membrane of monocytes/macrophages^70^. Human microglia constitutively express CD68 with higher expression levels in white than gray matter^39,45^. Microglial expression of CD68 is strongly induced by neuroinflammation^52,71^, which may also represent a tipping point in the pathogenesis of Alzheimer’s disease^72^. Interestingly, the brains of individuals resilient to dementia despite robust Alzheimer’s neuropathology (amyloid plaques and neurofibrillary tangles) displayed lower numbers of CD68^+^ microglia in the temporal lobe and higher levels of the cytokines IL-6, IL-1β, IL-10^73^. Recent evidence from genome-wide association studies suggests shared genetic architecture between MDD and late-onset Alzheimer’s disease^74,75^, and some of the identified genes were highly expressed in monocytes/macrophages and involved in immune response and endocytosis. However, the findings of our high-dimensional single-cell analysis of microglia in MDD do not lend support to neuroinflammatory changes in any of the examined cortical and subcortical brain regions. The results are in line with earlier studies of candidate markers like HLA-DR and CD68 in post-mortem brain tissue from MDD cases with and without suicidality^28–32^.

Our study has several limitations. The sample size is very small due to the difficulties in obtaining sufficient quality post-mortem brain tissue for CyTOF analysis. We were particularly interested in comparatively assessing different brain regions as human microglia are diverse, and we were able to replicate our earlier findings of regional heterogeneity of human microglia^39^ in this independent cohort. Although microglia from different post-mortem brain regions did not differ between MDD and controls, the attenuated expression of HLA-DR in microglia from the microenvironment of the subventricular zone may warrant further exploration. An important study has recently found that the brain transcriptional profile of MDD differs greatly by gender; men with MDD exhibited increases in oligodendrocyte- and microglia-related genes, while women with MDD had decreases in these markers^76^. Notably, *P2RY12* expression in the anterior cingulate cortex was increased in men with MDD and decreased in women with MDD compared to controls^76^. Our sample size was too small to correct for gender effects, but we did not observe different response patterns of microglia (including expression of P2Y_12_) between the three women and three men with MDD versus controls in our sample. Another confounder of our study may be the effects of medication, in particular antidepressants, on immune responses in the brain. It is well known that tricyclic antidepressants, selective serotonin reuptake inhibitors (SSRI), and serotonin and noradrenaline reuptake inhibitors (SNRI) can reduce the expression of proinflammatory cytokines like IL-6, IL-1β, and TNF-α, and increase the expression of IL-10, but the opposite effects have also been described^77^. Moreover, many of these studies examined peripheral blood and the cytokine changes may actually reflect treatment response. We cannot control for the effects of the diverse range of medication in our small sample, but it is important to note that the majority of MDD cases (at least 4/6) were clinically depressed at the time of death based on retrospective chart analysis and one case did not receive antidepressant medication during the last 3 months before death. Furthermore, 24 h before death, both control and MDD donors received morphine, opioids, sedatives and/or anesthetic agents (Supplementary Table 2). It has been demonstrated that these drugs may exert suppressive effects on the immune system and impair monocyte/macrophage function^78–80^. However, as a common limitation of studies performed in humans, the effects reported in the literature were variable and potentially confounded by different methods used to assess immune responses, large spectrum of drugs with different dosages, and low numbers of study participants^80^. In the future, it will be important to comparatively assess the effects of medication on peripheral immune cells and microglia in MDD by controlled trials. Finally, we were also unable to control for agony, comorbid conditions, and bias introduced by diagnosis based on retrospective medical chart review by two independent psychiatrists.

The results of this first high-dimensional single-cell analysis of microglia in MDD provide a missing piece in the concept of neuroinflammation in mood disorders. Further validation in larger cohorts and the use of additional techniques like single-cell RNA sequencing are required to ascertain the findings. Our results may be of particular value for PET studies which have relied on TSPO ligand binding to determine microglial responses in the CNS. Our findings also raise the intriguing possibility that supporting the functions of microglia in the brain may be more beneficial in MDD than the use of anti-inflammatory agents.

## Supplementary information

Supplementary Tables
