# Supplementary material for: Single-cell mass cytometry of microglia in major depressive disorder reveals a non-inflammatory phenotype with increased homeostatic marker expression
Source: Transl Psychiatry. 2020 Sep 11;10:310. doi: 10.1038/s41398-020-00992-2 (PMC7486938; doi:10.1038/s41398-020-00992-2)
Supplement: Supplementary file 1 — Supplementary Tables [file 41398_2020_992_MOESM1_ESM.pdf]

Supplementary Table 1 Donor and sample characteristics

Diagn.	Gender	Age (yr)	PMD (hr:min)	pH CSF	brain region	Data shown in Figures
CON-1	F	88	10:00	6.66	GFM<br>GTS<br>THA<br>SVZ	1 & 2
CON-2	F	55	7:30	NA	GFM<br>GTS<br>THA<br>SVZ	1 & 2
CON-3	F	83	6:50	6.75	GFM<br>GTS<br>SVZ	2
CON-4	F	82	5:30	6.48	GFM<br>THA<br>SVZ	2
CON-5	M	92	8:50	6.89	GFM<br>GTS<br>SVZ	2
MDD-1	M	61	5:45	NA	GFM<br>GTS<br>THA<br>SVZ	1 & 2
MDD-2	F	74	6:20	6.96	GFM<br>GTS<br>THA<br>SVZ	1 & 2
MDD-3	M	69	8:00	6.66	GFM<br>GTS<br>SVZ	2
MDD-4	F	23	8:35	NA	GFM<br>GTS<br>THA<br>SVZ	2
MDD-5	F	85	4:30	6.93	GFM<br>THA	2
MDD-6	M	48	12:45	6.76	GTS<br>SVZ	2
P value*	0.5455	0.1168	0.9581	0.2796	-	-

Diagn. = diagnosis; CON = control donor; MDD = major depressive disorder; F = female; M = male; Age (years); PMD = post-mortem delay (hours:minutes); CSF = cerebrospinal fluid; NA = not available; GFM = frontal lobe; GTS = temporal lobe; THA = thalamus; SVZ = subventricular zone.
* Fisher's exact test (Gender) and unpaired, two-tailed *t-test* (Age, PMD and pH CSF).

Supplementary Table 2 Donor medication history

Diagn.	Medication	
	**last 24 hours (drug and amount)**	**last 3 months (drug and dosage)**
CON-1	Furosemide (40 mg), Metoprolol (100 mg), Oxycodone (5 - 30 mg), Simvastatin (40 mg), Spironolactone (12.5 mg)	-
CON-2	Tramadol (50 – 200 mg)	Valproic acid (500 mg/day)
CON-3	Buprenorphine (5 µg/hour), Paracetamol (2000 mg), Prednisone (10 mg)	-
CON-4	Fentanyl patch (8.3 µg), Furosemide (40 mg), Hydroquinone (200 mg), Spironolactone (25 mg), Midazolam (5 mg), Modafinil (200 mg), Morphine (5 mg), Nebivolol (2.5 mg)	-
CON-5	Morphine (5 - 40 mg),	-
MDD-1	NA	Paroxetine (20 mg/day)
MDD-2	Thiopental (500 mg)	Amitriptyline (10 mg/day)
MDD-3	Midazolam (NA), Morphine (NA)	Oxazepam (24 mg/day)
MDD-4	Rocuronium bromide (NA), Sodium thiopental (NA)	Bupropion (300 mg/day), Imipramine (100 mg/day)
MDD-5	Barbiturate (NA), Esomeprazole (40 mg), Trimethoprim (100 mg), Zopiclone (7.5 mg), Calcium carbonate (500 mg)	Amitriptyline (25 mg/day), Mirtazapine (30 mg/day)
MDD-6	Propofol (NA), Rocuronium (150 mg), Thiopental (NA)	Tranylcypromine (40 mg/day)

Diagn. = diagnosis; CON = control donor; MDD = major depressive disorder; NA = not available

Supplementary Table 3 Psychiatric history of MDD cases

Donor	Duration of illness (years)	Depressive episodes	Severity
MDD-1	NA	recurrent episodes	moderate
MDD-2	40	single episode	moderate
MDD-3	37	recurrent episodes	mild
MDD-4	7	single episode	moderate
MDD-5	30	recurrent episodes	moderate
MDD-6	42	recurrent episodes	mild

NA = not available

Supplementary Table 4 List of antibodies and barcoding reagents for *Panel-A*

isotope tag	target	clone / company
^{141}Pr	CD45	HI30 / Fluidigm
^{142}Nd	CD19	HIB19 / Fluidigm
^{143}Nd	HLADR	L243 / Fluidigm
^{144}Nd	CD11b	ICRF44 / Fluidigm
^{145}Nd	CD18	TS1/18 / Fluidigm
^{146}Nd	CD64	10.1 / Fluidigm
^{147}Sm	CD11c	Bu15 / Fluidigm
^{148}Nd	CD16	3G8 / Fluidigm
^{149}Sm	CCL2	5D3-F7 / Biolegend
^{150}Nd	CCL4 (MIP-1β)	D21-1351 / Biolegend
^{151}Eu	CD68	Y1/82A / Biolegend
^{152}Sm	TNF	Mab11 / Fluidigm
^{153}Eu	CyclinB1	GNS-1 / Fluidigm
^{154}Sm	CD3	UCHT1 / Fluidigm
^{155}Gd	CD56	B159 / Fluidigm
^{156}Gd	CCR5 (CD195)	NP-6G4 / Fluidigm
^{158}Gd	CD101	BB27 / Fluidigm
^{159}Tb	IRF4	3E4 / Biolegend
^{160}Gd	CD163	GHI/61 / Biolegend
^{161}Dy	EMR1	A10 / Bio-Rad
^{162}Dy	Ki-67	B56 / Fluidigm
^{163}Dy	TGFβ	TW4-2F8 / Biolegend
^{164}Dy	CD115	9-4D2-1E4 / Biolegend
^{165}Ho	$P2Y_{12}$	polyclonal / Sigma-Aldrich
^{166}Er	IL-10	JES3-9D7 / Fluidigm
^{167}Er	IRF8	7G11A45 / Biolegend
^{168}Er	CD206	15-2 / Fluidigm
^{169}Tm	CD33	WM53 / Fluidigm
^{170}Er	CD86	IT2.2 / Biolegend
^{171}Yb	CCR2 (CD192)	K036C2 / Biolegend
^{172}Yb	CX3CR1	2A9-1 / Fluidigm
^{173}Yb	CD141	1A4 / Biolegend
^{174}Yb	CD32	6C4 / eBioscience
^{175}Lu	CD14	M5E2 / Fluidigm
^{176}Yb	TREM2	237920 / R&D Systems
^{191}Ir	DNA	- / Fluidigm
^{193}Ir	DNA	- / Fluidigm
^{209}Bi	CD47	CC2C6 / Fluidigm

Supplementary Table 5 List of antibodies and barcoding reagents for *Panel-B*

isotope tag	target	clone / company
^{141}Pr	HLA-DR	L243 / Fluidigm
^{142}Nd	CD116	4HI / Biolegend
^{143}Nd	IKZF1	Ikaros / Fluidigm
^{144}Nd	CD44	BJ18 / Biolegend
^{145}Nd	CD4	RPA-T4 / Biolegend
^{146}Nd	CD64	10.1 / Fluidigm
^{147}Sm	ALDH	44/ALDH / Fluidigm
^{148}Nd	Galanin	581403 / Fluidigm
^{149}Sm	CCL2	5D3-F7 / Biolegend
^{150}Nd	CD86	IT2.2 / Biolegend
^{151}Eu	CD68	Y1/82A / Fluidigm
^{152}Sm	CD95	DX2 / Biolegend
^{153}Eu	TIM3	F38-2E2 / Biolegend
^{154}Sm	CD172a	15-414 / Biolegend
^{155}Gd	CD54 (ICAM1)	HA58 / Biolegend
^{156}Gd	IL-6	29E.2A3 / Fluidigm
^{158}Gd	CD101	BB27 / Fluidigm
^{159}Tb	GM-CSF	BVD2-21C11 / Biolegend
^{160}Gd	CD14	RMO52 / eBioscience
^{161}Dy	TIM4	9F4 / Fluidigm
^{162}Dy	CD91	A2MR-α2 (RUO) / BD Bioscience
^{163}Dy	Glut5	195205 / R&D Systems
^{164}Dy	Arginase-1	658922 / Fluidigm
^{165}Ho	CD16	3G8 / Biolegend
^{166}Er	IL-10	JES3-9D7 / Fluidigm
^{167}Er	CCR7 (CD197)	G043H7 / Fluidigm
^{168}Er	CD130	2E1B02 / Fluidigm
^{169}Tm	-	-
^{170}Er	GPR56	CG4 / Biolegend
^{171}Yb	Glut1	195205 / R&D Systems
^{172}Yb	CX3CR1	2A9-1 / Fluidigm
^{173}Yb	CD141	1A4 / Biolegend
^{174}Yb	IL-1β	H1b-98 / Biolegend
^{175}Lu	TMEM119	HPA052650 / Abcam
^{176}Yb	MRP14	MRP 1H9 / Biolegend
^{191}Ir	DNA	- / Fluidigm
^{193}Ir	DNA	- / Fluidigm
^{209}Bi	CD11b	ICRF44 / Fluidigm
